# Explicating the Face Perception Network with White Matter Connectivity

**DOI:** 10.1371/journal.pone.0061611

**Published:** 2013-04-22

**Authors:** John A. Pyles, Timothy D. Verstynen, Walter Schneider, Michael J. Tarr

**Affiliations:** 1 Center for the Neural Basis of Cognition, Carnegie Mellon University and University of Pittsburgh, Pittsburgh, Pennsylvania, United States of America; 2 Department of Psychology, Carnegie Mellon University, Pittsburgh, Pennsylvania, United States of America; 3 Learning Research & Development Center, University of Pittsburgh, Pittsburgh, Pennsylvania, United States of America; 4 Department of Psychology, University of Pittsburgh, Pittsburgh, Pennsylvania, United States of America; University of Montreal, Canada

## Abstract

A network of multiple brain regions is recruited in face perception. Our understanding of the functional properties of this network can be facilitated by explicating the structural white matter connections that exist between its functional nodes. We accomplished this using functional MRI (fMRI) in combination with fiber tractography on high angular resolution diffusion weighted imaging data. We identified the three nodes of the core face network: the “occipital face area” (OFA), the “fusiform face area” (mid-fusiform gyrus or mFus), and the superior temporal sulcus (STS). Additionally, a region of the anterior temporal lobe (aIT), implicated as being important for face perception was identified. Our data suggest that we can further divide the OFA into multiple anatomically distinct clusters – a partitioning consistent with several recent neuroimaging results. More generally, structural white matter connectivity within this network revealed: 1) Connectivity between aIT and mFus, and between aIT and occipital regions, consistent with studies implicating this posterior to anterior pathway as critical to normal face processing; 2) Strong connectivity between mFus and each of the occipital face-selective regions, suggesting that these three areas may subserve different functional roles; 3) Almost no connectivity between STS and mFus, or between STS and the other face-selective regions. Overall, our findings suggest a re-evaluation of the “core” face network with respect to what functional areas are or are not included in this network.

## Introduction

What is the structure of the neural network supporting face recognition? Although the majority of research has focused on face-selective areas in isolation (e.g., the middle fusiform gyrus (mFus) or “FFA”) [1]–[3], more recent evidence reveals that face processing recruits multiple brain regions [4], [5] and appears to require a network of areas working in concert [6], [7]. Here we provide a more complete understanding of this network by considering anatomical structure, namely, the white matter tracts connecting these functionally-defined regions.

Over the past two decades, numerous face-selective regions other than the FFA have been reported (for review see: [8]), including a posterior ventral region labeled the “occipital face area” (OFA) [9]. While the great majority of neuroimaging research on face perception has focused on the FFA, a smaller number of studies have important reasons to investigate this area more closely (e.g.: [10]–[12]). Some evidence implicates OFA in lower-level facial feature processing as a precursor to more holistic processing in FFA [5], [13]. A less hierarchical view suggests that FFA responses may precede OFA responses [14]. Posterior superior temporal sulcus (STS) has also been found to be face selective [15], [16], likely supporting social information processing via the perception of eye gaze direction [17] and facial movement [16], [18]. The FFA, OFA, and STS have been called the “core” network for face perception [4], [19], [20].

More recently, growing evidence has also suggested a role for a region of the anterior inferior temporal lobe (aIT) that appears to support facial individuation [21]–[24]. Previous diffusion imaging studies using anatomical approaches have identified deficits in white matter pathways likely connecting fusiform to anterior temporal lobe regions in prosopagnosic and older populations, further bolstering the evidence for the important role of this area [7], [25].

The anatomical structure of the connections between functional areas also informs network function in the brain [26]. Diffusion imaging reveals white matter structure *in vivo*, and in combination with fMRI, can show the structural connectivity of functionally-defined networks. Combining high angular resolution diffusion spectrum imaging (DSI), generalized q-sampling reconstruction, and sub-voxel seeded deterministic tractography [27], [28], we use functionally-defined areas as seeds for fiber tracking, allowing us to precisely assess the structural connectivity between functional nodes of the core face network [29].

Using diffusion imaging to explore connectivity between face-selective regions, including the OFA, FFA/mFus, and STS, Gschwind et al. [30] found structural connectivity between the OFA and FFA, but not to STS – often considered part of the core network [4]. Similarly, using different tracking methods we find no direct connectivity between these regions and STS. We also find that the OFA should be divided into multiple anatomically distinct clusters – a re-characterization of what was previously identified as a single functional region. Finally, we observe structural connectivity between mFus and aIT, a region not functionally identified in previous diffusion imaging studies and thought to be of potentially critical importance in face perception. Together these results suggest a re-evaluation of connectivity within the face network as well as refinement of the putative functional roles of the specific network nodes.

## Methods

### Participants

Five right-handed healthy adults (1 female/4 male, mean age 28 years; range 22–33 years) participated in this study. Written informed consent was obtained from all participants prior to testing in accordance with procedures approved by the Institutional Review Boards of Carnegie Mellon University and the University of Pittsburgh. Participants were financially compensated for their time.

### fMRI Data Acquisition

Functional MRI data was collected in separate sessions from diffusion data acquisition with a 3T Siemens Verio MR scanner at the Scientific Imaging & Brain Research Center located on the Carnegie Mellon University campus using a 32-channel head coil. Functional images were acquired using a T2*-weighted echoplanar imaging (EPI) pulse sequence (31 oblique axial slices, in-plane resolution 2 mm×2 mm, 3 mm slice thickness, no gap, repetition time TR = 2000 ms, echo time TE = 29 ms, flip angle = 90°, GRAPPA = 2, matrix size = 96×96, field of view FOV = 192 mm). High-resolution anatomical scans were acquired for each participant using a T1-weighted MPRAGE sequence (1 mm×1 mm×1 mm, 176 sagittal slices, TR = 1870 ms, TI = 1100, FA = 8°, GRAPPA = 2).

### fMRI Stimuli and Procedures

Functionally-defined regions of interest (ROIs) selective for faces and places were identified using stimuli and procedures well-established in the field for these purposes, (e.g. [31], [32]). Face selective cortical areas were identified using a localizer scan consisting of alternating blocks of color photographs of faces and everyday objects. Place selective regions were identified using a scan with blocks of faces, places, objects, and scrambled objects. In both scans, blocks were 16 s in duration with 16 stimuli presented for 800 ms each with a 200 ms ITI and 6 s fixations between each block. A one-back identity task was used to maintain attention throughout both scanning sessions. Participants pushed a button on an MR compatible response glove when the current stimulus was the same as the preceding stimulus. Participants completed 2–4 runs of each localizer across two scanning sessions.

Stimuli were presented using Matlab (Mathworks, Natick, MA) and the Psychophysics Toolbox [33] controlled by an Apple Macintosh computer. Images were projected via a DLP projector (Sharp XG-P560W) through a wave guide into the scanner room onto a screen located at the head end of the bore, and viewed by the participant with a mirror attached to the head coil.

### fMRI Analysis

Preprocessing and analysis of fMRI localizer scans was performed in BrainVoyager QX 2.3 (Brain Innovations, Inc., Maastricht, The Netherlands). Functional data was 3D-motion corrected and temporally filtered, including linear trend removal and high-pass filtering using a GLM with Fourier basis set. The motion corrected data was then manually coregistered to the high-resolution anatomical data with the aid of high-resolution T2 weighted inplane scans, acquired in the same location as the functional slices, and manually checked for accuracy. No spatial smoothing was applied.

All fMRI analyses were performed in native brain space. Reported Talairach coordinates were determined after the analyses by applying a transformation to clusters center of mass coordinates to convert them from native participant space to Talairach space. ROIs were determined using standard general linear model analyses with predictors for each condition convolved with a canonical hemodynamic response function [34]. Face selective clusters were identified using the contrast faces>objects [2]. Place selective clusters were identified using the contrast places>objects [32]. All statistical maps were corrected for multiple comparisons using a false discovery rate (FDR) *q*<.05 [35].

### Diffusion Data Acquisition and Reconstruction

Diffusion data was acquired with a 3T Siemens Tim Trio MR scanner located at the University of Pittsburgh Medical Center using a 32-channel head coil. Participants were scanned with a 257 direction diffusion spectrum imaging (DSI) scan [36] using a twice-refocused spin-echo EPI sequence and multiple q-values with a 43 min acquisition time (TR = 9916 ms, TE = 157 ms, voxel size = 2.4×2.4×2.4 mm, FoV = 231×231 mm, b-max = 7000 s/mm2, 5 shells). DSI data were reconstructed using a generalized q-sampling imaging (GQI) approach [37] in DSI Studio (http://dsi-studio.labsolver.org). Orientation distribution functions (ODFs) were reconstructed to 362 discrete sampling directions and a mean diffusion distance scaling factor of 1.2. Due to low signal strength at high *b* values, no eddy current or head motion correction was applied.

### Fiber Tracking

Fiber tracking was performed using an ODF-streamlined, multi-FACT deterministic tractography algorithm [37]. All fiber tracking was performed in DSI Studio (http://dsi-studio.labsolver.org). Tractography was constrained by using functionally defined ROIs identified with the localizer scans (an approach similar to [29]). Since fiber streamlines tend to end near white matter/gray-matter boundaries, it is necessary for ROIs to include voxels that contain white matter in order for them to be used as ROIs for fiber tracking. Thus, functional ROIs were dilated by the equivalent of two diffusion data voxels in order to grow the regions into white matter. The dilated ROIs were then exported from BrainVoyager in NIfTI format. The high-resolution anatomical image coregistered to the fMRI data was then coregistered with a B0 or GFA image (whichever yielded the best co-registration result) from the DSI scan using SPM8 (Wellcome Department of Imaging Neuroscience, London, UK; www.fil.ion.ucl.ac.uk). The same transformation matrix was applied to the ROI NIfTI files and they were reoriented and re-sliced to the voxel and matrix dimensions of the DSI data using SPM8.

The coregistered ROIs were then used to constrain fiber tracking performed in DSI Studio. Tracking was performed between every possible pair of ROIs within a hemisphere using a whole brain seeding region. This allowed us to automatically save tracks that passed through both ROIs; all other tracks were discarded. Fibers were randomly seeded on a sub-voxel level, meaning that a fiber seed could have an initial position at any location within a diffusion data voxel. Tracking began at the random seed location in a random direction and continued in .5 mm steps. The direction of fiber progression was based on a weighting of diffusion data from both the current voxel and the surrounding voxels dependent on where the track point was located within the voxel. Thus the direction of fiber progression could vary within a voxel depending on the location of the seed. Directional momentum of streamlines was maintained by weighting the next directional estimate by 20%, and the previous direction by 80%. Tracking continued until the relative FA (fractional anisotropy) value of the incoming direction fell below a preset threshold (range 0.0241–0.0308, determined on an individual participant basis depending on relative signal-to-noise of each scan by thresholding so white matter voxels were above threshold and others below), or the upcoming turning angle exceeded a threshold of 80 degrees. This range of FA thresholds was consistent with previous studies using the same tractography procedures [28], [29], [38]–[40]. Fibers also had to fall within the length range of 20 mm to 140 mm, which represented a broad range of reasonable distances between two ROIs. The number of seeds used was determined on an individual participant basis by multiplying the number of voxels in a participant's whole brain mask in diffusion space by 1000, resulting in a seed number range of 113,586,000 to 139,786,000. Each resulting set of fibers between pairs of ROIs was saved in TrackVis format. These tracks were then imported into Matlab where custom scripts were used to eliminate any fibers that did not originate in one ROI and terminate in the other. Tracks were also manually inspected and any false tracks (e.g., anatomically implausible such as crossing a sulcus or passing through gray matter) were removed. Variance of track counts was tested by examining the track counts between mFus and other regions and was found to have unequal variance (Bartlett's test *p*<.0001), thus the track counts were log transformed before statistics were performed, improving homogeneity of variance (Bartlett's test *p* = .05).

## Results

### Face Selective Regions of Interest

**Figure 1:**
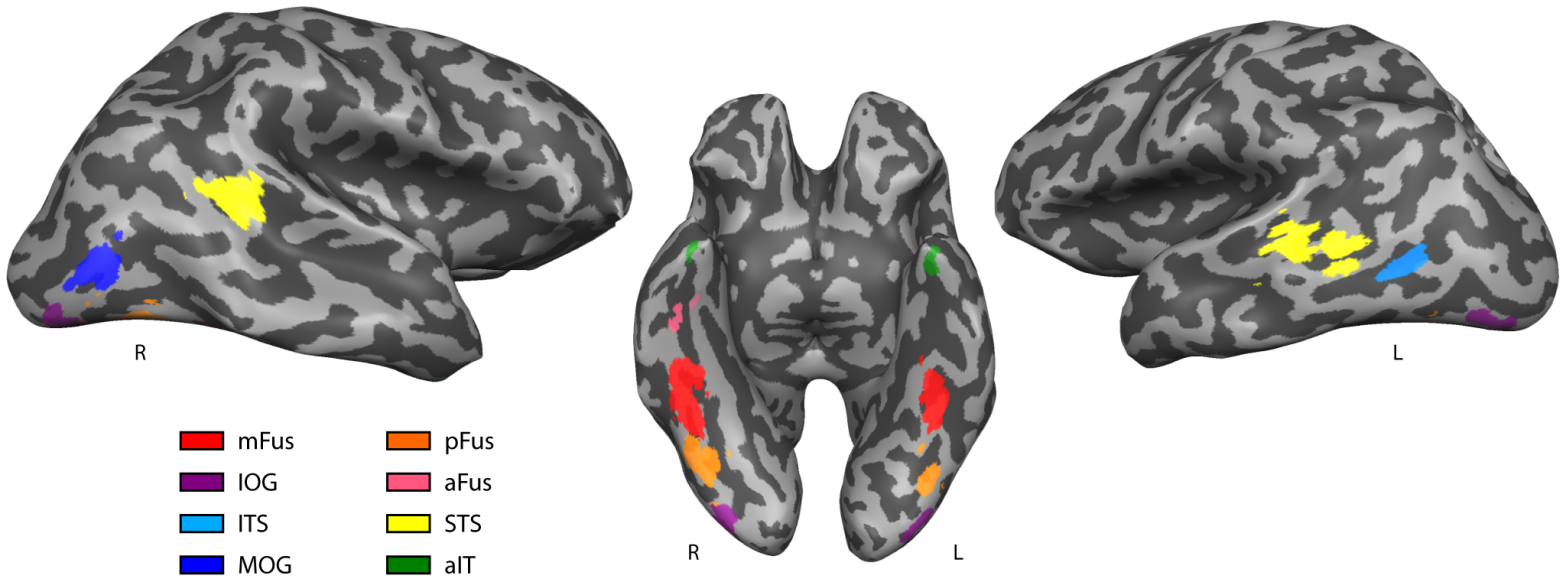
Face-selective regions of interest (ROIs). Regions of interest identified with fMRI using the contrast faces>objects in a representative participant thresholded at *q*<.05. Each region is shown on a partially inflated cortical surface in a unique color (see legend).

Multiple brain areas were found to be selective for faces using the standard functional localizer techniques employed here [20]. While only three areas are commonly reported and considered in occipito-temporal cortex, a larger number of spatially separable clusters likely exist in most individuals [41], [42]. Here we report up to 6 functionally-defined face selective regions in each participant's right and left hemispheres, shown in Figure 1.

#### mFus (“FFA”)

The most frequently reported face-selective brain area is a region of the middle fusiform (mFus) often referred to as the “fusiform face area” (FFA) [2]–[4]. A highly significant (*q*<.01) face-selective cluster was identified on the mFus in both hemispheres of all participants in the region typically labeled as FFA. If multiple clusters were present on the fusiform gyrus, the middle cluster closest to the typical coordinates of FFA was labeled as mFus.

#### Posterior Occipito-Temporal Regions (“OFA”)

A face-selective region posterior to the mFus/FFA cluster has been widely reported in the literature 9,43,44. While this region is commonly labeled the “occipital face area” (OFA) [9], the reported anatomical location varies substantially across studies [10], [42]. Our localizer scan results are consistent with recent observations of two face selective areas in occipito-temporal cortex where usually only a single OFA region was labeled [42]. The inferior occipital gyrus (IOG) is the region most often labeled OFA [45], however a region of the posterior fusiform gyrus (pFus) has also been identified as OFA [44]. The results of our localizer scans confirm all participants show at least two and sometimes three distinct face selective regions posterior to mFus. Instead of arbitrarily selecting one region to be labeled OFA, here we report *all* face-selective regions posterior to mFus by participant and label them according to their anatomical location (Table 1). In the right hemisphere, a face selective cluster on pFus was found in 2/5 participants, a cluster on the IOG in 5/5 participants, an inferior temporal sulcus (ITS) cluster in 4/5 participants, and a middle occipital gyrus (MOG) cluster in one participant. In the left hemisphere, 4/5 participants showed pFus clusters, 4/5 IOG clusters, 3/5 ITS clusters, and 2/5 a MOG cluster. No participant had both an ITS and an MOG region. Given their close anatomical proximity, these regions are likely analogous across participants and thus have been collapsed for the purposes of statistics. This level of cross-participant consistency in exhibiting face-selective regions posterior to the mFus is in line with the level of consistency seen in earlier studies [18].

**Table 1:** Face Selective Regions Talairach Coordinates (x, y, z).

	mFus	IOG	ITS	MOG	pFus	aFus	STS	aIT
*Right Hemisphere*								
Subject 1	(39, −40, −21)	(34, −80, −16)	*NA*	(52, −70, −2)	(40, −63, −16)	(43, −14, −25)	(53, −38, 9)	(35, 1, −38)
Subject 2	(38, −37, −20)	(44, −67, −13)	(46, −74, 14)	*NA*	*NA*	*NA*	(56, −50, 18)	(32, 0, −38)
Subject 3	(43, −52, −17)	(40, −82, −12)	(44, −75, −4)	*NA*	*NA*	(39, −33, −17)	(51, −38, 11)*	(36, −7, −25)
Subject 4	(35, −46, −18)	(39, −74, −20)	(39, −73, −4)	*NA*	*NA*	*NA*	(50, −38, 9)	(37, 2, −31)
Subject 5	(39, −48, −22)	(42, −78, −16)	(45, −58, −2)	*NA*	(34, −74, −23)	*NA*	(43, −44, 7)	(29, 2, −28)
*Left Hemisphere*								
Subject 1	(−38, −44, −20)	(−38, −84, −14)	(−40, −59, 2)	*NA*	(−34, −66, −16)	*NA*	(−58, −53, 4)	(−33, −5, −34)
Subject 2	(−34, −30, −25)	*NA*	(−39, −82, 7)	*NA*	(−42, −58, −21)	*NA*	(−47, −57, 13)	(−42, −5, −27)
Subject 3	(−36, −49, −18)	(−38, −88, −11)	*NA*	(−49, −82, 4)	*NA*	(−36, −34, −18)	(−56, −46, 9)*	(−37, −11, −27)
Subject 4	(−40, −37, −21)	(−34, −76, −23)	*NA*	(−45, −79, −5)	(−43, −54, −19)	*NA*	(−51, −40, 6)	(−43, −10, −20)
Subject 5	(−39, −46, −16)	(−39, −85, −7)	(−43, −77, 2)	*NA*	(−37, −65, −16)	*NA*	(−61, −49, 1)	(−30, −2, −29)

* Region did not pass FDR<.05 correction.

#### Superior Temporal Sulcus (STS)

A region of the posterior superior temporal sulcus (STS) has widely been reported as selective for faces, and STS is usually included as one of the three core face processing regions along with FFA and OFA [5]. A face selective region of STS was identified bilaterally in 4/5 of our participants. In one participant, a small bilateral STS cluster was present, but did not pass correction for multiple comparisons. In this participant, the small uncorrected STS cluster was dilated by 3 mm in order to be comparable in size to other participants' STS region for fiber tracking.

#### Anterior Temporal Lobe (aIT)

We identified a small face selective cluster in bilateral anterior inferior temporal lobe (aIT) in all of our participants. This is consistent with several studies that have identified a face selective region of the aIT [13], [21]–[24], [46]. While less commonly reported overall in the literature, this previous research suggests that aIT plays an important role in face individuation [5], [22], [24]. Sometimes labeled as a region in the “extended” face network [5], growing evidence suggests that aIT should be regarded as critical to face perception as the core STS, OFA and FFA regions [7]. While this cluster was smaller and weaker than the other face selective regions in our participants (likely due to its proximity to areas of poorer SNR due to susceptibility), it was significant (*q*<.05) and in the anatomical location reported by previous studies. Given the small size of these clusters, they were dilated by 3 mm in order to better match the volume of the other ROIs and compensate for likely effects of susceptibility at the far end of the temporal lobe pole. In the participant with the smallest clusters, the dilation was increased to 5 mm. In two participants, an additional region on the far anterior end of the fusiform gyrus was identified (one participant bilateral, one participant right hemisphere only). These clusters were spatially separate for the aIT clusters, and thus considered separate ROIs and labeled aFus.

### Structural white matter connectivity

#### Connectivity between mFus and occipital regions

**Figure 2:**
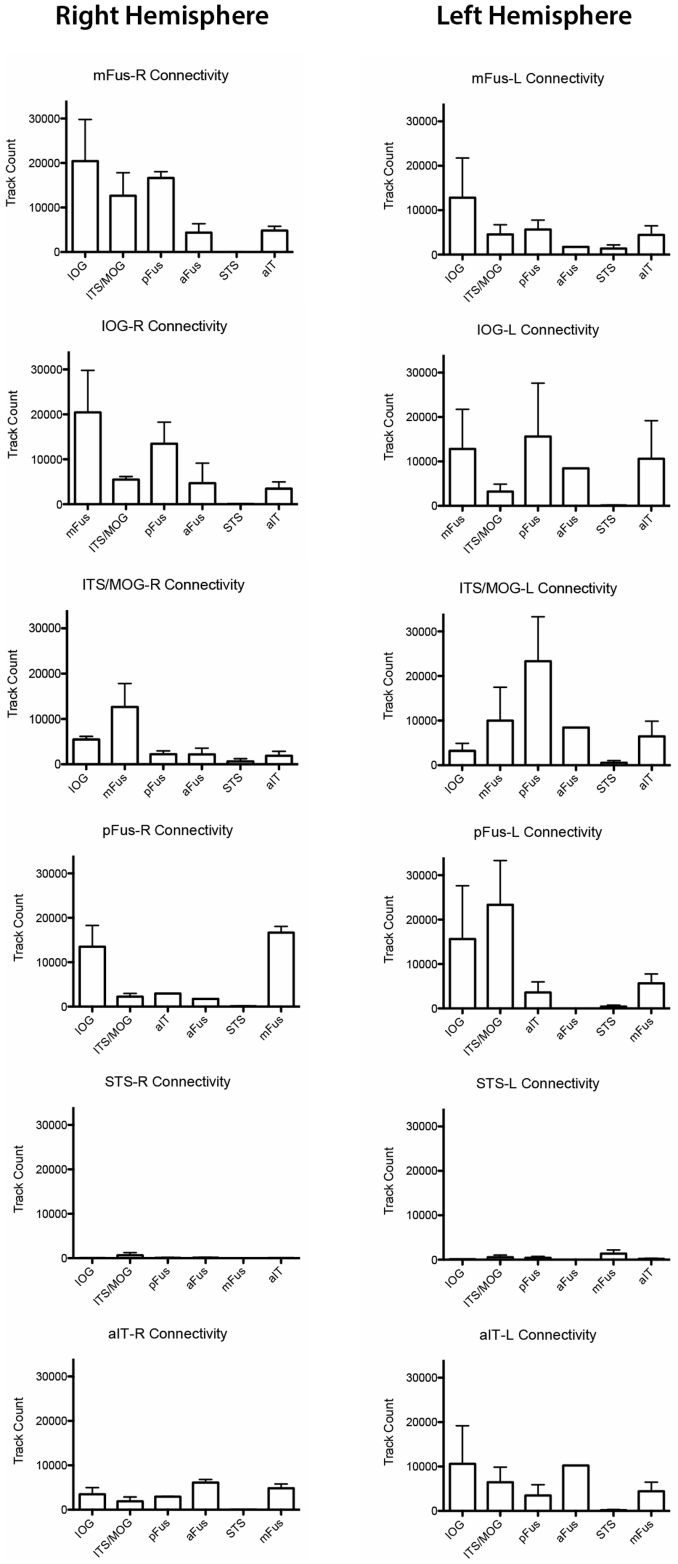
Connectedness of each major ROI to other ROIs. Each panel shows the track counts between one ROI and other ROIs. Error bars indicate standard error.

**Figure 3:**
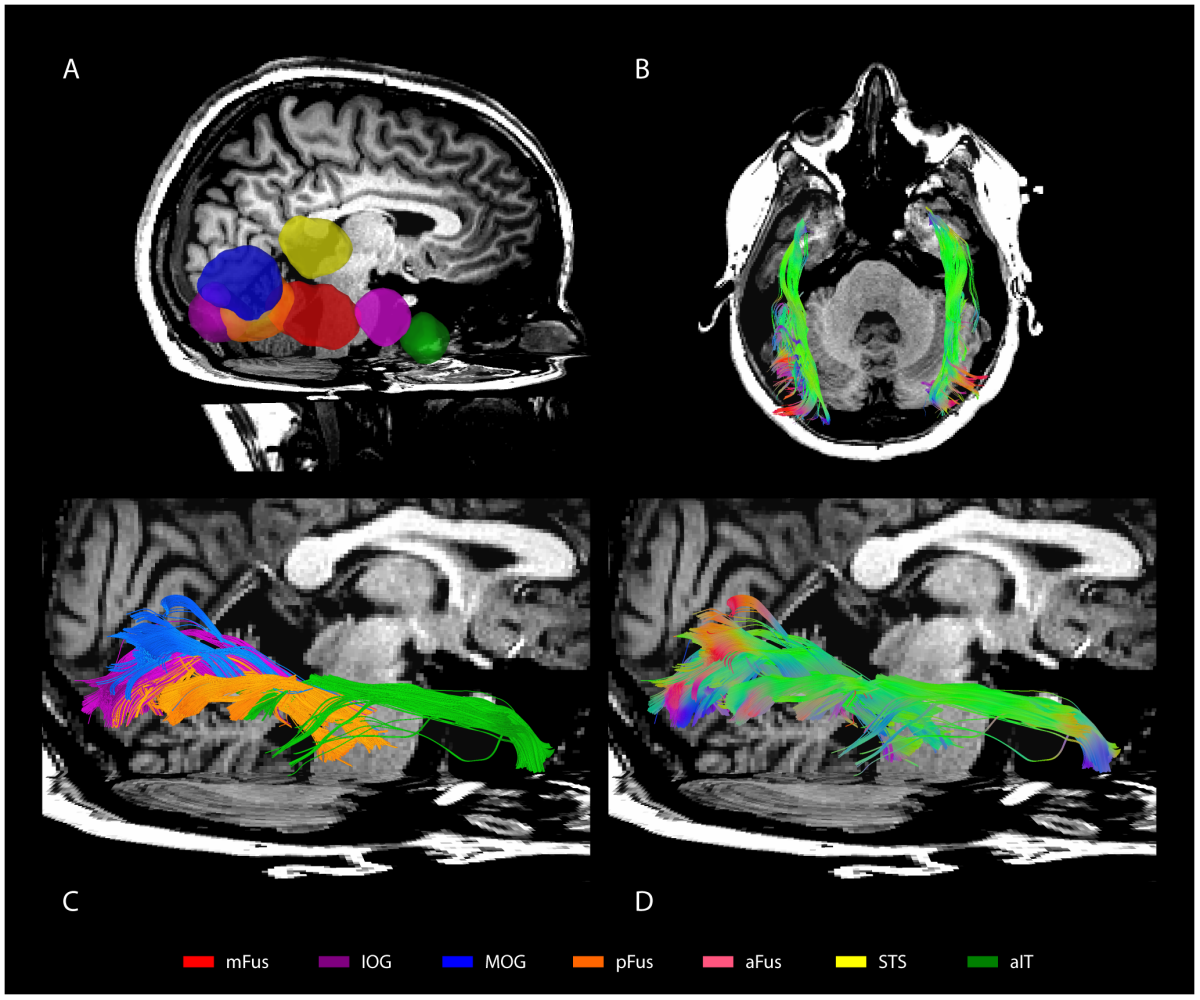
Fiber tracks connecting mFus to other ROIs in a representative participant. ***a*** **,** Expanded right-hemisphere ROIs rendered in 3D space with a high-resolution T1 anatomical co-registered to the diffusion data in the background. ***b*** **,** Fiber tracks connecting bi-lateral mFus to hemisphere respective ROIs shown from above. ***c*** **,** Fiber tracks connecting mFus-R to other ROIs with streamlines colored to correspond to the target ROI. ***d*** **,** The same fiber tracks as in ***c*** colored to indicate local directional information.

**Figure 4:**
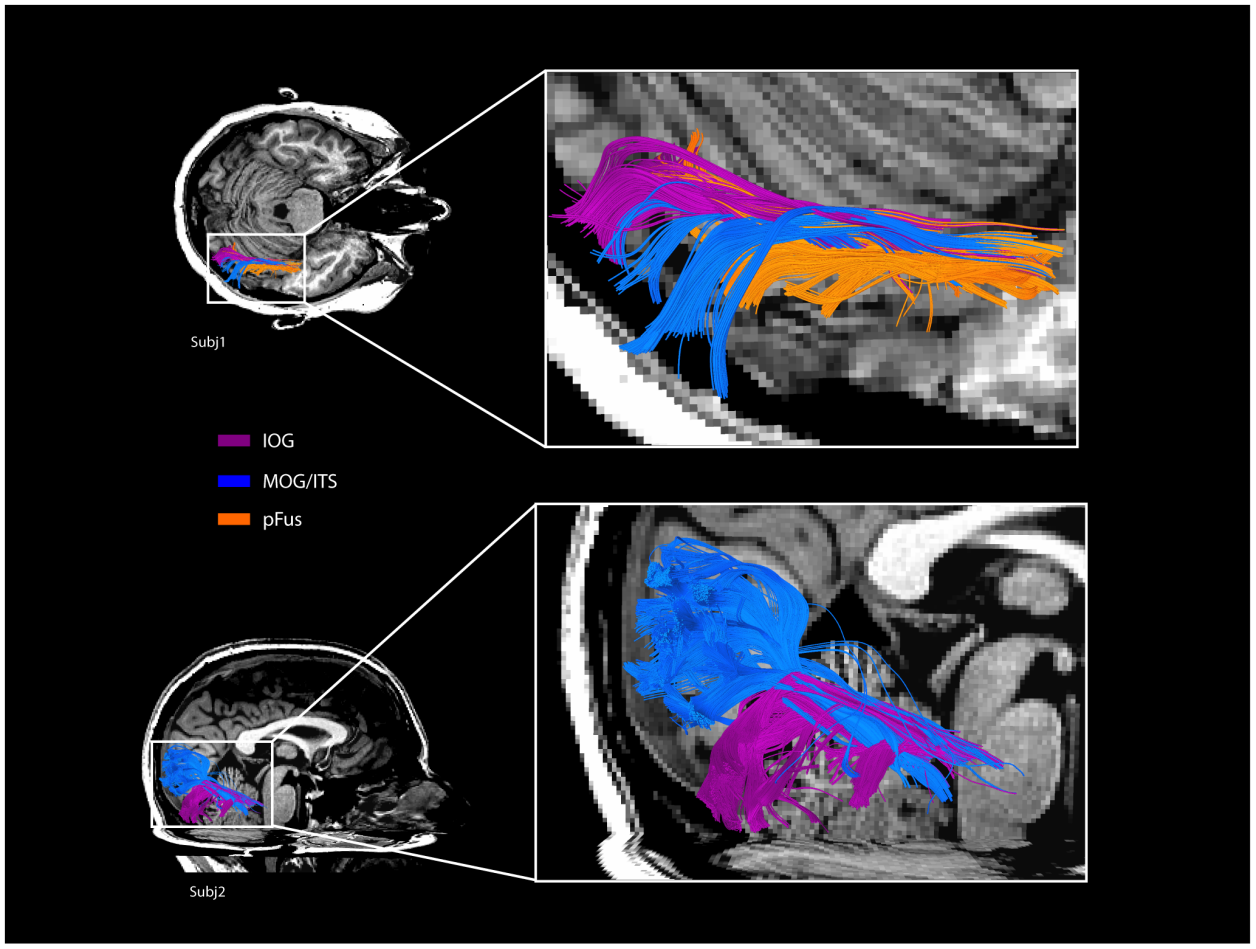
Fiber tracks connecting right mFus to occipital ROIs in two representative participants. Tracks are colored to correspond to their target ROI (see legend) and displayed from above (Subj1, top), and the side (Subj2, bottom).

Fiber tracks were found connecting mFus to all occipital ROIs in all participants (5/5) in the right hemisphere. Connectivity was strongest to the IOG region (Figure 2), the most consistent occipital face selective ROI in our participants and the most common anatomical location for the functional OFA area. Examples of fiber patterns to the multiple occipital regions are shown in Figure 3 and in detail in Figure 4. The three participants with pFus ROIs also showed connectivity to mFus. All participants also had a more dorsal face selective ROI that showed connectivity to mFus: 4/5 ITS, 1/5 MOG.

Connectivity of left mFus to left hemisphere occipital ROIs was also found for all ROI pairs and in all participants. The IOG again showed greatest connectivity, with pFus and more dorsal regions showing slightly less connectivity (Figure 2).

#### Connectivity to aIT

**Figure 5:**
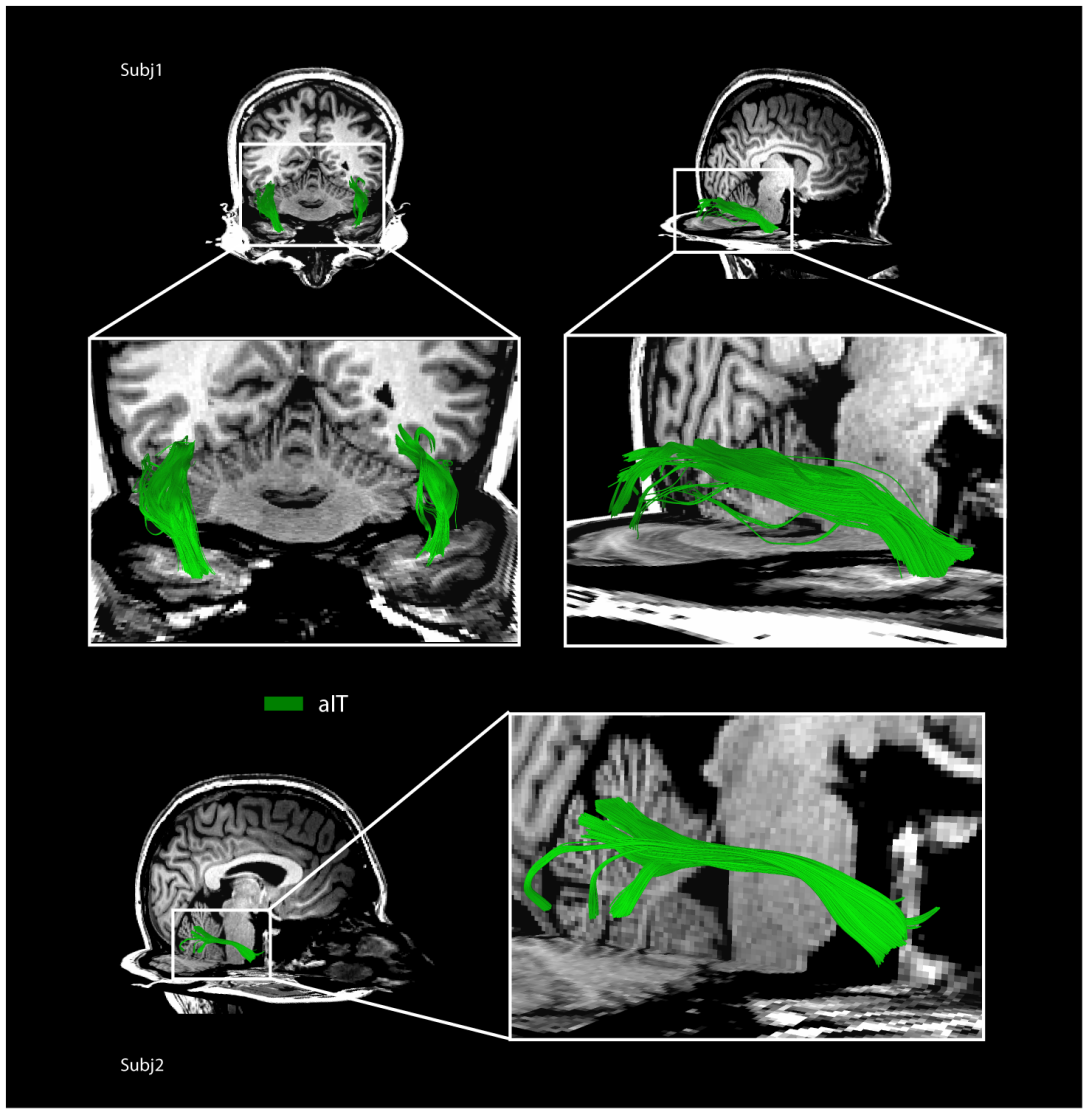
Fiber tracks connecting right mFus to aIT in two representative participants. In Subj1, two views are shown: partial bilateral tracks from above shown intersecting a coronal co-registered T1 anatomical slice (upper left), and the complete right track shown from the side (upper right). Note good correspondence between white matter in the T1 anatomical and the location of fiber tracks passing through the coronal slice. In Subj2 a complete right hemisphere track is shown from the side.

Connectivity was also found between the mFus region and aIT region in all participants (mean track count in both hemispheres = 4,365). While connectivity was lower to aIT than IOG and other occipital regions, it was consistent across participants, and fiber tracks showed anatomically plausible bundles of streamlines (Figure 5). Connectivity was also found between aIT and occipital regions, although to a lesser extent than mFus (Figure 2). This pattern of connectivity was the same in both left and right hemispheres for all participants.

#### Connectivity between occipital regions

In addition to long range connections between occipital areas and mFus and aIT, shorter range connections were also found between the occipital face selective areas. Connectivity was found between all possible pairs of the 2–3 occipital ROIs identified in each participant (Figure 2).

#### Lack of connectivity to STS

Almost no connectivity was found between right STS and mFus (mean track count = 21), with two participants showing *no* tracks between STS and mFus and the remaining three participants showing only a very small number of tracks (6, 11, and 89 tracks). These observations are supported statistically: connectivity from mFus to other regions was significantly greater than to STS (to IOG: *t*-test, *t* = 9.75, *p* = 0.0003, one-tailed; to ITS/MOG: *t*-test, *t* = 6.66, *p* = 0.0001, one-tailed; to aIT: *t*-test, *t* = 6.63, *p* = 0.001, one-tailed). Connectivity between STS and all other functional ROIs identified in our study was also almost non-existent (Figure 2). The occipital IOG, MOG and pFus ROIs all showed very little connectivity, and the same was true for aIT. Almost no connectivity was present between STS and ITS in three participants with this ROI, with the fourth showing some connectivity (2,966 tracks).

Connectivity between left STS and left mFus was more variable than in the right hemisphere (mean track count = 1,652), with two participants showing no tracks, one participant showing very few (103 tracks), and two showing moderate connectivity (3,661 and 4,494 tracks). However, overall connectivity between mFus and STS was less than mFus to other areas, significant in ITS/MOG (*t*-test, *t* = 2.31, *p* = 0.04, one-tailed) and marginally significant in IOG (*t* = 2.16, *p* = 0.06, one-tailed), but not significant in aIT (*t* = 1.48, *p* = 0.11, one-tailed).

#### Connectivity to a control area

**Figure 6:**
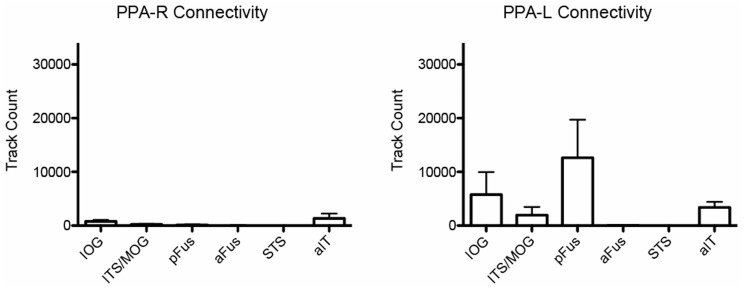
Connectedness (measured by track counts) of the PPA control area to other ROIs. Error bars indicate standard error.

Robust connectivity between functionally-defined regions is only diagnostic if one can also establish a *lack* of connectivity between other candidate regions. That is, we must be sure that these methods do not simply demonstrate that “everything is connected to everything.” While we have already identified a node of the face network that lacks connectivity to other face selective regions (STS), below we show an additional control analyses that suggests that our connectivity results are meaningful. In order to control for the possibility that there is substantial connectivity between *all* areas of ventral occipito-temporal cortex, we performed tracking between a functionally-defined control region *not* implicated in face perception and face-selective ROIs. We chose the parahippocampal place area (PPA) as our control ROI since it has often been used as a control region to contrast mFus/FFA in fMRI studies of face perception [26], [47], [48], and its neuroanatomical location is very close to the mFus cluster. PPA was identified bi-laterally in all participants by contrasting images of scenes with objects in the fMRI localizer scan described above (*q*<.05). Tracking was then performed between PPA and all face-selective ROIs. In the right hemisphere, connectivity from mFus to occipital IOG and ITS/MOG ROIs was significantly greater than connectivity from PPA to the same ROIs (*t*-tests: to IOG: *t* = 4.95 , *p* = 0.004, one-tailed; to ITS/MOG: *t* = 3.5 , *p* = 0.003, one-tailed). Connectivity of mFus to the pFus ROI was observed in 6/10 hemispheres and was also greater than connectivity of PPA to pFus (*t-*test, t = 5.05, p = .004, one-tailed). Connectivity from mFus to aIT was also greater than from PPA to aIT, (*t*-test, *t* = 3.02, *p* = 0.02, one-tailed). In the left hemisphere, mean track counts were lower between PPA and IOG, ITS/MOG, and aIT as compared to mFus, but not significantly so (*t*-tests: to IOG: *t* = 1.15 , *p* = 0.17, one-tailed; to ITS/MOG: *t* = 1.31 , *p* = 0.13, one-tailed; to aIT: *t* = −0.07 , *p* = 0.53, one-tailed). Mean track count across the four participants with this ROI to pFus was higher for PPA than to FFA (*t*-test: *t* = −0.64, *p* = .71, one-tailed) (Figure 6).

#### Overlap with ILF and IFOF

**Figure 7:**
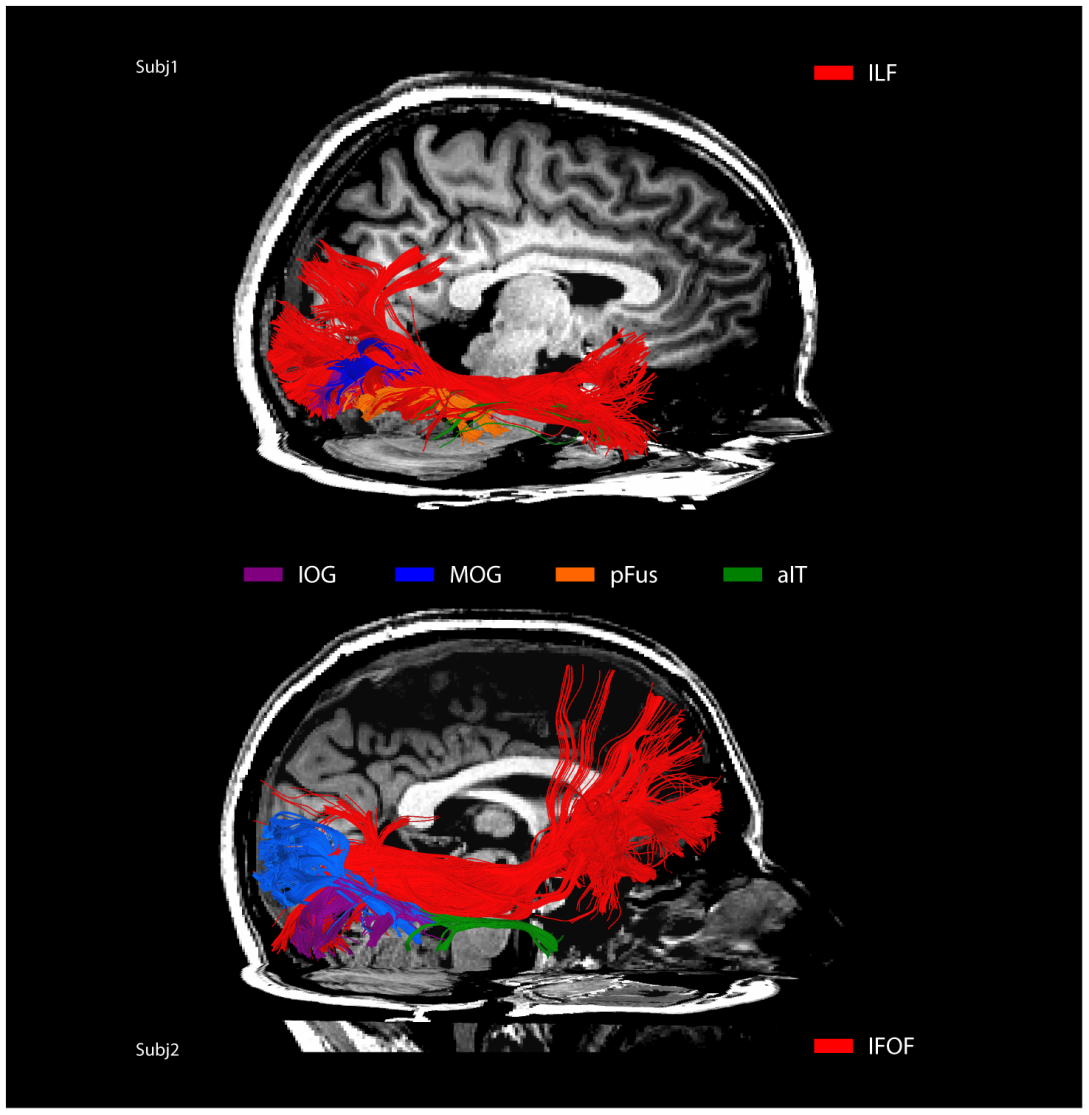
Spatial overlap of right ILF and IFOF with mFus connected tracks in two representative participants. The major ILF and IFOF tracts are colored red, and the mFus connected tracks are colored to correspond with their target ROIs. IFOF is show in the top participant and ILF in the bottom participant.

Prior studies using diffusion imaging to study face-perception networks identified reduced FA values in the inferior longitudinal fasciculus (ILF) and the inferior fronto-occipital fasciculus (IFOF), two major white matter tracts in ventral cortex running anterior to posterior [7], in congenital prosopagnosics compared to normal controls. To relate our present findings to these earlier results, we identified the ILF and IFOF using the same anatomical ROI approach as Thomas et al. [7] and compared the resulting spatial locations of these two tracks to the tracks identified in our study. We observed substantial spatial overlap between the ILF and IFOF and the tracks connecting face-selective regions (Figure 7): across all voxels that contained fibers between face-selective regions, 51% (SD = 9%) of these voxels overlapped with voxels that contained ILF fibers, and 19% (SD = 10%) overlapped with voxels that contained IFOF fibers. A large number of the anterior to posterior bundles we identified fell within the ILF and IFOF, especially tracks connecting pFus and IOG to FFA, and FFA to aIT. Bundles connecting to aIT were particularly coincident with the ILF. More posterior fiber bundles connecting occipital areas with FFA were less clearly spatially aligned with the ILF and IFOF, however both of these tracts show considerable fanning of fibers within occipital lobe, thus it is more difficult to assess overlap.

#### Hemispheric differences

Although the overall pattern of global connectivity found in this study is similar across hemispheres, with all of the same qualitative connections and lack of connections found between face-selective ROIs, from a quantitative perspective we do see evidence of hemispheric differences. Consistent with this observation, in a diffusion study of connectivity between functionally-identified face-selective regions, Gschwind et al. [30] found a higher probability of OFA-FFA connectivity in the right hemisphere relative to the left hemisphere. However, it is important to note that this difference is quantitative, not qualitative: based on Figure 4 of [30] there was a connectivity probability of approximately 0.5 between right OFA-FFA and approximately 0.25 between left OFA-FFA; both probabilities are much higher than almost any other probabilities measured in their study. Our present results replicate this quantitative effect in that a comparison of right hemisphere OFA-FFA connectivity versus left hemisphere connectivity revealed differences between the two hemispheres. However, although we observe higher track counts on the right, these differences did not reach significance (paired *t*-tests: IOG: *t* = 1.90, *p* = 0.08, one-tailed; ITS/MOG: *t* = 1.62 , *p* = 0.09, one-tailed). Other ROIs also did not show significant differences across hemispheres (paired *t*-tests: STS: *t* = −1.70 , *p* = 0.08, one-tailed; aIT: *t* = .93 , *p* = 0.20, one-tailed). Some hemispheric differences were also found in tracking to PPA and STS, and are described above.

## Discussion

**Figure 8:**
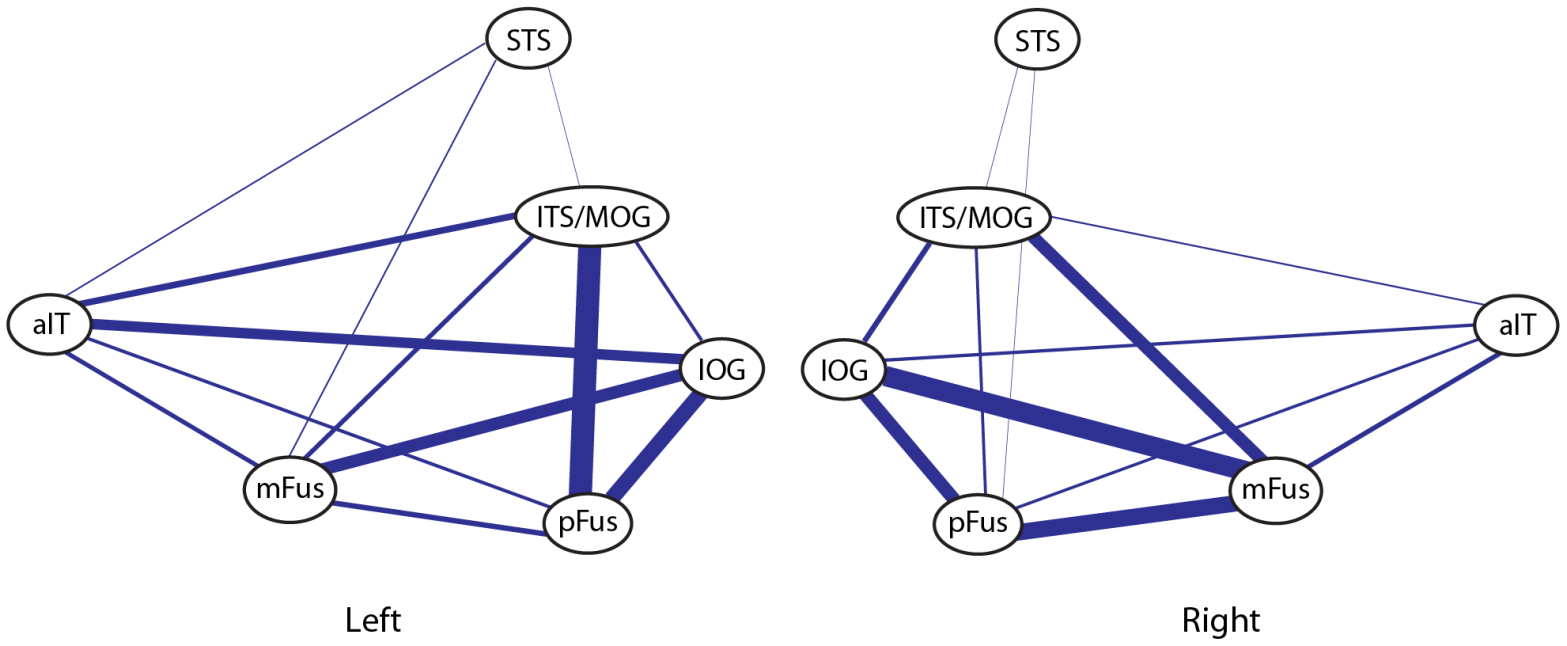
Connectivity diagram showing connections between all face-selective ROIs in both hemispheres. Line thickness is scaled to reflect the mean track count across all participants. The aFus ROI is not shown as it was only present in 3 of 10 hemispheres.

A combination of diffusion imaging methods and functional MRI allowed us to characterize the structural connectivity of the cortical network for face processing *in vivo*, showing the connectivity pattern of the core network, as well as an additional face selective area, aIT (Figure 8). We find strong connectivity between the mFus face-selective area and more posterior brain regions that comprise two critical nodes of the putative core face processing network. However we did not find consistent structural connections to STS, the nominal third node of this network. For the first time, we also identified connectivity between mFus and occipital regions to a functionally defined face selective region of aIT, a region recently identified as playing an important role in face recognition. *In toto*, our results reveal a connectivity pattern that is somewhat different from that typically assumed to underlie the face-processing network, suggesting the possible addition of aIT in any account of a core network, and a reevaluation of the role of STS given its lack of direct connectivity.

Our finding of white matter connectivity between mFus and the posterior OFA regions is consistent with previous findings linking processing between these areas [10]. However, the majority of studies tend to identify one OFA cluster in analyses. In contrast, growing evidence suggests that the spatial location of this single reported OFA area varies considerably, and that most participants likely have at least two separable face-selective clusters in occipital cortex posterior to mFus [10], [42]. The results of our functional localizer scans confirm this observation, as we found 2–3 face-selective regions in occipital cortex in all participants. Our tracking results showed connectivity between all of these posterior areas to mFus. The functional selectivity and consistent structural connectivity of these different occipital regions implicates them all in face perception, presumably subserving different computations that interact with processing in the mFus face-selective region.

While IOG was the most common anatomical location for a posterior face selective cluster in both the present study and past findings [10], [49], the IOG was not necessarily the area most strongly connected to mFus across participants. Rather, the posterior face-selective region with the largest number of fibers to mFus varied by participant, reflecting possibly individual differences that should be behaviorally assessed [23]–[25], [49].

Connectivity was also found between the posterior face-selective areas themselves. We suggest that the 2–3 posterior face-selective regions found in all participants form a “complex” or sub-network much like the LOC, where information is exchanged and the processing role varies between regions. While the anatomical locations of second and third areas varied across participants (IOG being the consistent posterior area), the spatial arrangement of one area being more dorsal and one more ventral was consistent, with participants either having a pFus and IOG area, or an IOG and ITS/MOG area. This is analogous to the LOC, which normally shows a more ventral cluster(s) on the ventral surface (often labeled pFus) and a more superior cluster on the lateral surface (often labeled LO) [50].

The finding of structural connectivity between all of the posterior face-selective areas and the mFus suggests that more attention should be paid to careful identification of distinct posterior face-selective regions, in that they may possibly serve different computational roles. Thus further investigation using neuroimaging combined with other approaches is warranted.

Our finding of white matter connectivity to the face selective aIT region provides further evidence for the importance of this area in face perception. Recent fMRI studies have provided accumulating evidence that aIT is crucial for the task of identifying faces and might possibly support some of the computations previously attributed to the FFA [23], [24]. Other diffusion imaging results also potentially point to an important role of more anterior areas in face processing. Most relevant are the findings of Thomas et al. [7], who found, in individuals with congenital prosopagnosia, reduced structural connectivity along major white matter tracks running posterior to anterior in ventral cortex. While these individuals showed normal fusiform activation using fMRI, they had impaired face recognition. The reduced structural connectivity of the ILF and IFOF is an indication that their deficit in face recognition is the result of reduced connectivity of mid and posterior ventral occipito-temporal cortex to more anterior regions. Here we identified connectivity between both the mFus and occipital face selective regions to aIT, and also showed that these tracks spatially overlap with ILF and IFOF, and are potentially sub-tracks of these major bundles. Given the evidence for the functional role of aIT, and association between reduced ILF and IFOF connectivity and impairment in face processing, it seems likely that critical information for face individuation is transmitted through these white matter pathways.

In contrast, given its previously attributed role as a core region of the face network, connectivity would be expected to STS. However, we found few if any connections between STS and all other ROIs, which is evidence that STS may not be part of the core face perception network. Instead, STS may play a functionally-distinct role in face perception from other nodes of the network, and thus lack connectivity to them. Evidence from fMRI studies supports this possibility: STS has been shown to be involved in aspects of face perception for which more ventral areas do not appear to be recruited, for example, eye gaze and facial motion [16], [51], [52]. Our confidence in the lack of structural connectivity for the STS to other face-selective regions is reinforced by its consistency with the results reported by Gschwind et al. [30], who also observed, using a somewhat different diffusion imaging pipeline, a similar absence of white matter connections to STS. Of course, this lack of structural connectivity does not rule out functional connectivity between STS and other face network regions which could be mediated by additional regions. However these results are also somewhat mixed, with studies both supporting functional connectivity between STS and mFus [53], and those finding little functional connectivity between STS and mFus [54], [55].

The neuroanatomical location of STS places it in the dorsal processing stream, as opposed to mFus/FFA and IOG/OFA's locations in the ventral stream. We speculate that the differing functional roles of STS and its lack of structural connectivity in comparison to these other areas may reflect this larger separation of processing pathways. Instead of directly exchanging information with the other nodes of the network, STS may be receiving information through separate white matter pathways, as well as through gray matter. For example, given the selectivity of STS to motion in both faces and bodies [16], [56], [57], information could be passed to STS from the generally motion selective hMT+ complex earlier in the dorsal stream. However, further studies will be required to determine the connectivity of STS more precisely.

In that our present results reveal a more refined connectivity pattern in the right hemisphere that is more weakly reflected in the left hemisphere, our findings are also consistent with the neural processing of faces being right lateralized [2], [58], [59]. Our speculation is that the robust face-selective neural responses reliably observed in the right hemisphere arise in part because such selectivity is enabled by the underlying structural connectivity of the right ventral visual pathway [60].

While diffusion imaging is currently the only method to investigate white matter pathways using neuroimaging data *in vivo*, limitations of this technique should be considered when interpreting tractography results. Tractography does not provide data indicating the directionality of information along white matter pathways. So while diffusion imaging can provide information about the structural architecture of a cortical network, an overall account including information flow will require complimentary methodologies that provide good temporal information about the time course of activity in the brain such as MEG and EEG. Fiber tracking also has limitations in resolving complex fiber crossing and turns dictating caution in interpretation of a finding of lack of connectivity. However, we are confident in our results here regarding STS given that our methodology uses DSI, which is more resistant to the crossing problem [61], and that our results are in line with those reported by Gschwind et al. [30] using different diffusion methodologies.

We also acknowledge that our study has fewer individual subjects than some other recent diffusion imaging studies. With respect to the quality of our data, it should be noted that our DSI sequence uses 257 directions and five shells, which provides improved estimates of water diffusion that enables more accurate deterministic tractography. This sort of measurement is analogous to increasing power through an increased number of observations per subject, as opposed to increasing the number of subjects with fewer observations. Moreover, because the ODF model used with DSI provides more information than standard DTI methods, we have more information about diffusion directions and strengths within each voxel. Exemplifying the power of this approach, several recent papers relying on the same methods used here have revealed new understanding about structural connectivity in the human attention [29] and motor systems [28], [39].

In contrast, most studies with larger numbers of subjects utilize DTI sequences with a smaller number of directions (commonly 12–64), which reduces the quality of the tractography. The tradeoff here is our diffusion imaging methods require a dedicated session for the 45 min DSI scan with experienced subjects to minimize motion and additional sessions for fMRI – here a total of 2–3 scanning sessions per a subject – while the majority of other studies employ much shorter DTI scans in conjunction with fMRI within the same, single scanning session. Consistent with this sort of tradeoff, as mentioned above, many other recent studies utilizing high angular-direction scans such as HARDI or DSI have comparable numbers of subjects to our current study, ranging from 4–6 in total [28], [29], [62], [63], or, in one instance, only showing single subject results [64]. Perhaps more importantly, in terms of interpreting the reliability of our present results, what is critical is that across *all* of our subjects we observed a consistent connectivity pattern that leads to our three main findings. These considerations leave us confident that our results and their interpretation are based on sufficient measurement and statistical power, here expressed in terms of the observations per participant, and would not qualitatively change with additional subjects.

We used a combination of fMRI and diffusion imaging methods to characterize the white matter structural connectivity of the network of cortical areas associated with face perception. Our findings speak to several questions regarding the structural connectivity of the core face perception network and reinforce the importance of an additional area: aIT. First, using fMRI, we are able to more precisely separate the traditional “OFA” region into 2–3 anatomically distinct clusters, and then, using DSI, demonstrate structural connectivity between these functional regions and middle fusiform gyrus. That each of these occipital regions is connected to mFus, as well as to each other, suggests that these regions may subserve different functional roles within a larger functional complex. Notably, very few other studies have separated “OFA” into functional subregions [42]. Second, we observe little connectivity between STS and other face-selective regions. This structural separation of STS from other face-selective regions is consistent with findings that STS activity is more functionally distinct than the closely-related occipital, mid-fusiform, and anterior temporal regions. Third, we identified structural connectivity between functionally-localized regions of aIT and the mFus with occipital face-selective areas – a result that is consistent with earlier diffusion imaging that did not employ functionally-identified areas, as well as newly emerging accounts of the face perception network where aIT plays a critical role [7], [24]. We believe that this is the first time that structural connectivity between the functionally-defined aIT and mFus has been demonstrated. In sum, our findings provide substantial new information about the potential computational roles and structural connectivity underlying the human cortical face processing network, suggesting that a re-characterization of the traditional face network model may be in order.
